# Serum levels of soluble platelet endothelial cell adhesion molecule-1 and vascular cell adhesion molecule-1 are decreased in subjects with autism spectrum disorder

**DOI:** 10.1186/2040-2392-4-19

**Published:** 2013-06-17

**Authors:** Yosuke Kameno, Keiko Iwata, Hideo Matsuzaki, Taishi Miyachi, Kenji J Tsuchiya, Kaori Matsumoto, Yasuhide Iwata, Katsuaki Suzuki, Kazuhiko Nakamura, Masato Maekawa, Masatsugu Tsujii, Toshirou Sugiyama, Norio Mori

**Affiliations:** 1 Department of Psychiatry, Hamamatsu University School of Medicine, 1-20-1, Handayama, Higashi-ku, Hamamatsu, Shizuoka 431-3192, Japan; 2 Research Center for Child Mental Development, University of Fukui, 23-3 Matsuokashimoaizuki, Eiheiji, Fukui 910-1193, Japan; 3 Department of Pediatrics, Nagoya City University Hospital, 1-Kawasumi, MIzuho, Mizuho-ku, Nagoya 467-8602, Japan; 4 Research Center for Child Mental Development, Hamamatsu University School of Medicine, 1-20-1 Handayama, Higashi-ku, Hamamatsu, Shizuoka 431-3192, Japan; 5 Department of Neuropsychiatry, Hirosaki University School of Medicine, 5-Zaifu,Hirosaki, Aomori 036-8562, Japan; 6 Department of Laboratory Medicine, Hamamatsu University School of Medicine, 1-20-1 Handayama, Higashi-ku, Hamamatsu, Shizuoka 431-3192, Japan; 7 Schoolof Contemporary Sociology, Chukyo University, 101 Tokodachi, Kaizu, Toyota, Aichi 470-0393, Japan; 8 Department of Child and Adolescent Psychiatry, Hamamatsu University School of Medicine, 1-20-1 Handayama, Higashi-ku, Hamamatsu, Shizuoka 431-3192, Japan

**Keywords:** Autism, Human serum, Adhesion molecules, Platelet-endothelial adhesion molecule-1, Platelet selectin, Endothelial selectin, Intracellular adhesion molecule-1, Vascular cell adhesion molecule-1

## Abstract

**Background:**

Adhesion molecules, such as platelet-endothelial adhesion molecule-1 (PECAM-1), platelet selectin (P-selectin), endothelial selectin (E-selectin), intracellular adhesion molecule-1 (ICAM-1), and vascular cell adhesion molecule-1 (VCAM-1), are localized on the membranes of activated platelets and leukocytes and on the vascular endothelium. Recently, we measured serum levels of soluble (s) forms of adhesion molecules in adults,18 to 26 years old, with autism spectrum disorder (ASD) and observed low levels of sPECAM-1 and sP-selectin. A subsequent study showed a similar result in children two to four years old with ASD. However, information about school age (five to seventeen years old) ASD subjects is required to determine whether adhesion molecules are also reduced in individuals with ASD in this age range.

**Findings:**

Twenty-two subjects with high-functioning ASD and 29 healthy age-matched controls were recruited. ELISA was used for sPECAM-1, and a suspension array system was used for sP-selectin, sE-selectin, sICAM-1 and sVCAM-1 measurements. We found that serum levels of sPECAM-1 (*U* = 91.0, *P*<0.0001 by Mann–Whitney *U* test) and sVCAM-1 (*U* = 168.0, *P* = 0.0042) were significantly lower in ASD subjects than in controls. Subsequently, we examined the correlations between serum levels of either sPECAM-1 or sVCAM-1 and clinical variables including Autism Diagnostic Interview - Revised subscores and our previous cytokine profile data from the same ASD subjects. However, we did not find any significant correlations between them.

**Conclusions:**

The present results, taken together with previous results, suggest that sPECAM-1 may play a role in the generation and development of ASD, beginning in childhood and lasting until adulthood.

## Findings

### Background

Autism spectrum disorder (ASD) is a developmental disorder that is characterized by severe impairment in social interaction and communication and by the presence of stereotyped behavior. The mechanisms underlying the pathophysiology of this disorder remain to be determined [1,2]. However, accumulating evidence suggests that the immune system plays a role in the pathophysiology of ASD [3-5].

Adhesion molecules, such as platelet-endothelial adhesion molecule-1 (PECAM-1), platelet selectin (P-selectin), endothelial selectin (E-selectin), intracellular adhesion molecule-1 (ICAM-1) and vascular cell adhesion molecule-1 (VCAM-1) are localized on the membranes of activated platelets and leukocytes and on the vascular endothelium [6,7]. They mediate the binding of leukocytes to the blood vessel wall, which is the main step in the process of inflammation [6,7]. Soluble PECAM-1 exists in a 90 kDa cleaved form and a 120 kDa form produced by mRNA alternative splicing, both of which are present at approximately equal levels in plasma [8].We previously measured serum levels of soluble (s) forms of the adhesion molecules in adults (18 to 26 years of age) with high-functioning ASD [9,10]. We found that the levels of sPECAM-1 and sP-selection were significantly decreased in ASD adults compared with controls [9,10], while sVCAM-1 showed a weak trend towards a lower level [10]. Children with ASD (two to four years of age) have significantly lower levels of sPECAM-1 and sP-selectin compared with controls [11]. Taken together, these results suggest that adhesion molecules may be involved in the pathophysiology of ASD. However, serum levels of adhesion molecules in school-age (five to seventeen years of age) ASD subjects have not been examined. Moreover, it is still unclear how they relate to immune abnormalities or inflammatory phenotypes of ASD. In the present study, we measured serum levels of sPECAM, sP-selectin, sE-selectin, sICAM-1 and sVCAM-1 in males five to seventeen years of age, with high-functioning ASD. A recent systemic serum proteome profiling study reported that males and females with Asperger’s disorder have distinct biomarker fingerprints [12]. It has been argued that genetic variation for ASD and intellectual disability cluster in genes involved in distinct pathways and protein complexes [13]. Therefore, to prevent any potential confounding effects of sex and intelligence, we recruited only high-functioning males. Subsequently, we tested the relationships between serum levels of adhesion molecules and clinical variables in high-functioning ASD subjects. Additionally, we examined the correlation between adhesion molecules and the significantly-altered cytokine profile of this population according to our previous cytokine profile data [14].

### Methods

#### Ethics approval

This study received approval from the ethics committee of the Hamamatsu University School of Medicine. All participants gave written informed consent before voluntary enrollment.

### Subjects

Twenty-two male subjects with high-functioning ASD (five to seventeen years of age) and 29 age-matched (eight to sixteen years of age) and healthy male control individuals were included in this study. All the participants were Japanese, born and living in the Aichi, Gifu or Shizuoka Prefectures of central Japan.

Based on interviews and available records, including those from hospitals, the diagnosis of autism was made based on the Diagnostic and Statistical Manual, fourth edition, text revision (DSM-IV-TR) criteria. The Autism Diagnostic Interview-Revised (ADI-R) was also conducted by two of the authors (KJT and KM), who are experienced and reliable at diagnosing autism with the Japanese version of the ADI-R [15]. ADI-R is a semi-structured interview conducted with a parent, usually the mother, and is used to confirm the diagnosis and evaluate the core symptoms of autism [15]. We also used the Wechsler Intelligence Scale for Children, third edition (WISC-III), to exclude subjects with a full-scale IQ of less than 70. Comorbid psychiatric illnesses were excluded using the Structured Clinical Interview for DSM-IV (SCID). Participants were excluded from the study if they had any symptoms of inflammation, a diagnosis of autoimmune disease, fragile X syndrome, epileptic seizures, obsessive-compulsive disorder, affective disorder, or any additional psychiatric or neurological diagnosis. All the autistic subjects were drug-naive and were not taking any dietary supplements.

Healthy control subjects were recruited locally by advertisement. All control subjects underwent a comprehensive assessment of their medical history to eliminate individuals with neurological or other medical disorders. SCID was conducted to scrutinize any personal or family history of past or present mental illness. None of the control subjects initially recruited fulfilled any of these exclusion criteria.

### Sampling and assay

Serum samples were collected as described previously [9,10]. Serum levels of sPECAM-1/CD31 were determined using the appropriate commercially available sandwich ELISA kits (Instant ELISA, eBioscience, San Diego, CA, USA) according to the manufacturer’s instructions. Serum levels of the other adhesion molecules were assayed with a suspension array system (Bio-Plex; BioRad, Hercules, CA USA), using a Fluorokine MAP Multiplex Human Adhesion Molecule Panel (R&D Systems Inc., Minneapolis, MN, USA), including human sE-selectin/CD62E, sP-selectin/CD62P, sICAM-1/CD54, and sVCAM-1/CD106. Multiplex kits for measuring cytokines including IL-1β, IL-1RA, IL-5, IL-8, IL-12(p70), IL-13, IL-17 and growth-related oncogene (GRO)-α were purchased from Bio-Rad (Bio-Plex Pro Human Cytokine Group I (27-plex) and Group II (21-plex) panels). This system allows simultaneous identification of adhesion molecules with antibodies chemically attached to fluorescently labeled micro-beads. The beads were resuspended in assay buffer and the reaction mixture was quantified using a Bio-Plex protein array reader. The sera of all subjects with high-functioning ASD were measured together in one assay with a set of control sera. Each serum sample was analyzed in duplicate, and the mean value of the two measures was used for the analyses. Concentrations (pg/ml) of different analytes in the serum samples were determined by using standard curves generated in the multiplex assays. Each standard curve was generated using eight points of concentrations, and a nonlinear least squares minimization algorithm was used for the curve fitting by the 5PL equation which determines the high and low limits of detection. To exclude inflammatory disease, serum C-reactive protein (CRP) levels were determined using a routine clinical biochemistry automatic analyzer.

### Performance characteristics of the immunoassays

#### Sensitivity

The limit of detection of the assays, defined as the analyte concentration resulting in an absorbance significantly higher than that of the dilution medium (mean plus 2 standard deviations), was determined to be 0.06 ng/ml (human sPECAM-1), 2.4 pg/ml (human sICAM-1), 4.2 pg/ml (human sP-Selectin), 0.55 pg/ml (human sE-Selectin), and 1.72 pg/ml (human sVCAM-1).

#### Reproducibility

Reproducibility within the assay was evaluated in three independent experiments. Each assay was performed with six replicates of eight serum samples containing different concentrations of the analyte. The calculated overall intra-assay coefficient of variation was 7.0% (human sPECAM-1), 6.2% (human sICAM-1), 6.1% (human sP-Selectin), 5.3% (human sE-Selectin), and 5.2% (human sVCAM-1). The calculated overall inter-assay coefficient of variation was 4.2% (human sPECAM-1), 17.7% (human sICAM-1), 12.7% (human sP-Selectin), 13.4% (human sE-Selectin), and 14.7% (human sVCAM-1).

#### Recovery

The spike recovery was evaluated by spiking four levels of human sPECAM-1 into human serum. Recoveries were determined in three independent experiments with six replicates each. The unspiked serum was used as a blank in these experiments. The average recovery ranged from 94% to 121% with an overall mean recovery of 107%. In the suspension array system, cell culture media samples were spiked with a recombinant analyte and evaluated for recovery. The average recovery was 99% (human sICAM-1: range 83 to 112%), 104% (human sP-Selectin: range 85 to 118%), 104% (human sE-Selectin: range 95 to 113%), and 98% (human sVCAM-1: range 85 to 112%).

### Statistical analysis

Clinical characteristics (age, weight, height, body mass index (BMI) and CRP) were analyzed using an unpaired *t* test, after confirmation that there were no significant differences in variance as assessed by the *F* test. Comparisons of concentrations of sPECAM-1, sE-selectin, sP-selectin, sICAM-1, and sVCAM-1 between ASD subjects and controls were made using the Mann–Whitney *U* test because of significant differences in variance as assessed by the *F* test. Evaluation of the relationships between levels of both sPECAM-1 and sVCAM-1 and clinical variables or symptom profiles was performed using Spearman’s rank correlation coefficient. Values of *P*<0.05 were considered significant. All statistical analyses were performed using SPSS software (version 18.0 J; IBM, Tokyo, Japan).

### Results and discussion

The characteristics of all the participants are summarized in Table 1. There were no significant differences in the distributions of age, weight, height and BMI between the subjects with high-functioning ASD and the controls. There were also no significant differences in CRP between the high-functioning ASD and the control groups.The CRP measurement of one subject with ASD was 2.30 mg/dl (this individual did not have subjective symptoms or a history of inflammatory disease). There were no significant differences in a full-scale IQ of WISC-III.

**Table 1:** Clinical characteristics of control individuals and subjects with high-functioning ASD

**Group**	**Control (number = 29)**	**ASD (number = 22)**	***P*-value**
Age, years	11.6 ± 1.9 (8 to 16)	11.5 ± 3.2 (5 to 17)	NS
Weight, kg	40 ± 9.9 (24 to 57.6)	41.4 ± 16.5 (17.5 to 96.6)	NS
Height, cm	149.3 ± 11.9 (121.4 to 167)	147 ± 18.3 (110 to 178)	NS
BMI, kg/m^2^	17.7 ± 2.3 (14.4 to 25.3)	18.5 ± 3.5 (13.9 to 30.5)	NS
CRP, mg/dl	0.02 ± 0.02 (0.01 to 0.08)	0.13 ± 0.44 (0.01 to 2.30)	NS
ADI-R			
Domain A score	N/A	20.7 ± 5.0 (10 to 27)	
Domain BV score	N/A	14.2 ± 4.2 (8 to 21)	
Domain C score	N/A	5.3 ± 2.0 (3 to 9)	
Domain D score	N/A	3.2 ± 1.0 (2 to 5)	
Sum	N/A	43.5 ± 9.6 (28– to 0)	
WISC-III			
Full-scale IQ	103.5 ± 13.1 (82 to 129)	97.2 ± 17.7 (72 to 134)	NS
Adhesion molecules			
sPECAM-1 (ng/ml)	77.8 ± 13.2 (41.9 to 99.3)	58.4 ± 12.5 (36.4 to 82.4)	<0.0001
sE-Selectin (micro g/ml)	44.8 ± 13.2 (24.1 to 89.7)	42.9 ± 19.1 (13.1 to 93.2)	NS
sP-Selectin (micro g/ml)	69.5 ± 21.2 (38.9 to 125.5)	78.1 ± 28.9 (39 to 137.5)	NS
sICAM-1 (micro g/ml)	325.8 ± 41.5 (243.8 to 422.1)	323.3 ± 56.4 (223.5 to 428.2)	NS
sVCAM-1 (micro g/ml)	916.5 ± 106.6 (749.4 to 1160.8)	810.5 ± 146.8 (561.8 to 1162.5)	0.0042

Values are expressed as mean ± s.d. (range). *ADI-R* Autism Diagnostic Interview-Revised, *ASD* autism spectrum disorder, *BMI* body mass index, *CRP* C-reactive protein, *ICAM-1* intracellular adhesion molecule-1, *IQ* intelligence quotient, *N/A* not applicable, *NS* not significant, *PECAM-1* platelet-endothelial adhesion molecule-1, *s* soluble, *VCAM-1* vascular cell adhesion molecule-1, *WISC-III* Wechsler Intelligence Scale for Children, Third Edition.

The serum levels of sPECAM-1 in subjects with high-functioning ASD were significantly lower than those of controls (*U* = 91.0, *P*<0.0001) (Table 1). Subjects with high-functioning ASD also had significantly decreased levels of sVCAM-1 compared with those in controls (*U* = 168.0, *P* = 0.0042) (Table 1). There were no significant differences in sE-selectin, sP-selectin, and sICAM-1 levels between high-functioning ASD subjects and controls (*U* = 298.0, *P* = 0.6966 for sE-selectin; *U* = 268.0, *P* = 0.3369 for sP-selectin; and *U* = 302.0, *P* = 0.9144 for sICAM) (Table 1).

We then examined the correlations between serum levels of either sPECAM-1 or sVCAM-1 and clinical variables among ASD subjects. Neither sPECAM-1 nor sVCAM-1 levels had significant correlations with clinical variables, including age, weight, height, BMI, CRP, ADI-R subscale scores and full-scale IQ (data not shown). We determined that plasma concentrations of IL-1β, IL-1RA, IL-5, IL-8, IL-12(p70), IL-13, IL-17 and GRO-α were significantly higher in subjects with ASD compared with the corresponding values of the matched controls, after correcting for multiple comparisons [14]. Subsequently, we examined the correlations between serum levels of either sPECAM-1 or sVCAM-1 and our previous cytokine profile data from the same ASD subjects. However, we did not find any significant correlations between them (Table 2). Our finding of low serum levels of sPECAM-1 and sVCAM-1 in the present study provides further support of previous reports that adhesion molecules may have a role in the pathophysiology of ASD, possibly by causing abnormalities in the immune system [14,16]. Similar to our subjects with ASD (five to seventeen years of age), children (two to four years of age) [11] and adults (18 to 26 years of age) [10] with ASD also have low levels sPECAM-1. However, sVCAM-1 levels are not altered in ASD children [11], but there is a weak trend towards lower levels in ASD adults [10]. Our results of reduced levels of sVCAM-1 in the current study appear to be compatible with those obtained in ASD adults, who show a tendency towards a decrease in sVCAM-1 levels. Therefore, we propose that sVCAM-1 may play a limited role in the pathophysiology of ASD. These findings suggest that sPECAM-1 may play an important role in the generation and development of the pathophysiology of ASD, beginning in childhood and lasting until adulthood. PECAM-1 has been implicated as an important mediator for the transendothelial migration of leukocytes *in vivo* and *in vitro*[17]. However, the requirement for PECAM in the transmigration of leukocytes is not absolute [18]. Rather, the absence of PECAM may facilitate the migration of leukocytes across the blood–brain barrier, presumably due to compensation by other adhesion molecules [19]. For instance, during progression of experimental autoimmune encephalomyelitis (a model for multiple sclerosis), mononuclear cell extravasation and infiltration of the brain in PECAM-1-deficient mice are considerably enhanced [18]. Additionally, cultured PECAM-1-deficient endothelial cells in mice exhibit prolonged changes in response to histamine treatment [18]. Recently, our measurements with positron emission tomography showed that adults with high-functioning ASD have significantly increased activated microglia in a wide range of brain areas [20]. Decreased sPECAM-1 levels might facilitate the infiltration and accumulation of leukocytes in the brain, leading to an increase in activated microglia.

**Table 2:** Correlation between the adhesion molecules and cytokines of control individuals and subjects with high-functioning ASD

			**Control**			**ASD**		
**Analytes**			**CC**	**number**	***P*-value**	**CC**	**number**	***P*-value**
sVCAM-1								
	IL-1β		0.009	29	NS	0.383	22	NS
	IL-1RA		−0.265	29	NS	0.055	22	NS
	IL-5		−0.158	29	NS	0.011	22	NS
	IL-8		−0.159	29	NS	0.281	22	NS
	IL-12	(p70)	−0.050	28	NS	0.333	22	NS
	IL-13		−0.240	29	NS	0.144	22	NS
	IL-17		0.195	10	NS	0.215	16	NS
	GRO-α		−0.256	25	NS	0.269	21	NS
sPECAM-1								
	IL-1β		0.102	29	NS	−0.100	22	NS
	IL-1RA		−0.056	29	NS	−0.067	22	NS
	IL-5		−0.134	29	NS	0.126	22	NS
	IL-8		−0.063	29	NS	−0.205	22	NS
	IL-12	(p70)	0.132	28	NS	0.008	22	NS
	IL-13		0.227	29	NS	−0.106	22	NS
	IL-17		−0.109	10	NS	−0.056	16	NS
	GRO-α		−0.136	25	NS	−0.064	21	NS

*ASD* autism spectrum disorder, *CC* correlation coefficient, *GFO-α* growth-related oncogene α, *NS* not significant, *PECAM-1* platelet-endothelial adhesion molecule-1, *s* soluble, *VCAM-1* vascular cell adhesion molecule-1.

When we tested the correlation between levels of either sPECAM-1 or sVCAM-1 and clinical variables including ADI-R subscores and serum levels of ILs, no overt interrelationship was observed. In this test, we were focusing on IL-8 and GRO-α, in particular, because both are chemokines produced by macrophages and other cell types, such as epithelial and endothelial cells. These chemokines have chemotactic activity on neutrophils and play an important role in the innate immune response. Previous studies have examined IL-8 levels in plasma or serum and found either increased [21] or no change [12,22] in peripheral IL-8 levels. The reason why these chemokines are increased in subjects with ASD is currently unknown [14]. However, our result suggests that reduced levels of sPECAM-1 and sVCAM-1 are not involved in the clinical aspects or IL alterations of school-aged boys with ASD. These alterations of adhesion molecules may be caused by events besides a known inflammation signaling in subjects with ASD. In contrast, Onore *et al*. observed significant associations between PECAM-1 levels and higher repetitive behavior scores in ASD children [11]. It is, therefore, of interest to examine the correlation between serum levels of PECAM-1 and interleukins in very young children with and without autism to clarify how these adhesion molecules in serum may reflect their central function associated with clinical aspects of ASD.

There are limitations to the present study. The small sample size renders the data preliminary, and a larger study with more subjects with ASD is necessary. However, recruitment for the current study was limited to a group of high-functioning male subjects with ASD (these subjects were not given psychotropic drugs). Therefore, our data are free from confounding factors due to sex, intelligence and psychotropic drugs and, thus, reflect a certain common immunological pathology among people with ASD. Moreover, the samples were measured in duplicate. Although triplicate measures were recommended to exclude any spurious measurements, in the present study we could not ensure that there were enough serum samples for triplicate measurements.

## Abbreviations

ADI-R: Autism diagnostic interview-revised; ASD: Autism spectrum disorder; BMI: Body mass index; CRP: C-reactive protein; DSM-IV-TR: Diagnostic and statistical manual, fourth edition, text revision; ELISA: Enzyme-linked immunosorbent assay; E-selectin: Endothelial selectin; GRO: Growth-related oncogene; ICAM-1: Intracellular adhesion molecule-1; IL: Interleukin; P-selectin: Platelet selectin; s: Soluble; SCID: Structured clinical interview for DSM-IV; VCAM-1: Vascular cell adhesion molecule-1; WISC-III: Wechsler intelligence scale for children, third edition.

## Competing interests

The authors declare that they have no competing interests.

## Authors’ contributions

HM, KI and NM designed the study. KS, KN, MT and TS were involved in the recruitment of participants. HM, TM and KN collected blood samples. KJT and KM conducted clinical evaluations. YK, HM and YI measured and analyzed serum levels of the adhesion molecules. MM measured and analyzed serum levels of CRP. YK, KI, HM and NM participated in manuscript preparation. All authors read and approved the final manuscript.

## References

[B1] Baron-Cohen S Belmonte MK Autism: a window onto the development of the social and the analytic brain Annu Rev Neurosci 2005 28 109 126 10.1146/annurev.neuro.27.070203.144137 16033325

[B2] Volkmar FR Pauls D Autism Lancet 2003 362 1133 1141 10.1016/S0140-6736(03)14471-6 14550703

[B3] Cohly HH Panja A Immunological findings in autism Int Rev Neurobiol 2005 71 317 341 16512356 10.1016/s0074-7742(05)71013-8

[B4] Krause I He XS Gershwin ME Shoenfeld Y Brief report: immune factors in autism: a critical review J Autism Dev Disord 2002 32 337 345 10.1023/A:1016391121003 12199139

[B5] Pardo CA Vargas DL Zimmerman AW Immunity, neuroglia and neuroinflammation in autism Int Rev Psychiatry 2005 17 485 495 10.1080/02646830500381930 16401547

[B6] Lee SJ Benveniste EN Adhesion molecule expression and regulation on cells of the central nervous system J Neuroimmunol 1999 98 77 88 10.1016/S0165-5728(99)00084-3 10430040

[B7] Blankenberg S Barbaux S Tiret L Adhesion molecules and atherosclerosis Atherosclerosis 2003 170 191 203 10.1016/S0021-9150(03)00097-2 14612198

[B8] Goldberger A Middleton KA Oliver JA Paddock C Yan HC DeLisser HM Albelda SM Newman PJ Biosynthesis and processing of the cell adhesion molecule PECAM-1 includes production of a soluble form J Biol Chem 1994 269 17183 17191 8006026

[B9] Iwata Y Tsuchiya KJ Mikawa S Nakamura K Takai Y Suda S Sekine Y Suzuki K Kawai M Sugihara G Matsuzaki H Hashimoto K Tsujii M Sugiyama T Takei N Mori N Serum levels of P-selectin in men with high-functioning autism Br J Psychiatry 2008 193 338 339 10.1192/bjp.bp.107.043497 18827301

[B10] Tsuchiya KJ Hashimoto K Iwata Y Tsujii M Sekine Y Sugihara G Matsuzaki H Suda S Kawai M Nakamura K Minabe Y Yagi A Iyo M Takei N Mori N Decreased serum levels of platelet-endothelial adhesion molecule (PECAM-1) in subjects with high-functioning autism: a negative correlation with head circumference at birth Biol Psychiatry 2007 62 1056 1058 10.1016/j.biopsych.2006.12.018 17509538

[B11] Onore CE Nordahl CW Young GS Van de Water JA Rogers SJ Ashwood P Levels of soluble platelet endothelial cell adhesion molecule-1 and P-selectin are decreased in children with autism spectrum disorder Biol Psychiatry 2012 72 1020 1025 10.1016/j.biopsych.2012.05.004 22717029 PMC3496806

[B12] Schwarz E Guest PC Rahmoune H Wang L Levin Y Ingudomnukul E Ruta L Kent L Spain M Baron-Cohen S Bahn S Sex-specific serum biomarker patterns in adults with Asperger's syndrome Mol Psychiatry 2011 16 1213 1220 10.1038/mp.2010.102 20877284

[B13] Kou Y Betancur C Xu H Buxbaum JD Ma'ayan A Network- and attribute-based classifiers can prioritize genes and pathways for autism spectrum disorders and intellectual disability Am J Med Genet C Semin Med Genet 2012 160C 130 142 10.1002/ajmg.c.31330 22499558 PMC3505691

[B14] Suzuki K Matsuzaki H Iwata K Kameno Y Shimmura C Kawai S Yoshihara Y Wakuda T Takebayashi K Takagai S Matsumoto K Tsuchiya KJ Iwata Y Nakamura K Tsujii M Sugiyama T Mori N Plasma cytokine profiles in subjects with high-functioning autism spectrum disorders PLoS One 2011 6 e20470 10.1371/journal.pone.0020470 21647375 PMC3103577

[B15] Tsuchiya KJ Matsumoto K Yagi A Inada N Kuroda M Inokuchi E Koyama T Kamio Y Tsujii M Sakai S Mohri I Taniike M Iwanaga R Ogasahara K Miyachi T Nakajima S Tani I Ohnishi M Inoue M Nomura K Hagiwara T Uchiyama T Ichikawa H Kobayashi S Miyamoto K Nakamura K Suzuki K Mori N Takei N Reliability and validity of Autism Diagnostic Interview-Revised, Japanese Version J Autism Dev Disord 2013 43 643 662 10.1007/s10803-012-1606-9 22806002

[B16] Rossignol DA Frye RE A review of research trends in physiological abnormalities in autism spectrum disorders: immune dysregulation, inflammation, oxidative stress, mitochondrial dysfunction and environmental toxicant exposures Mol Psychiatry 2012 17 389 401 10.1038/mp.2011.165 22143005 PMC3317062

[B17] Muller WA The role of PECAM-1 (CD31) in leukocyte emigration: studies in vitro and in vivo J Leukoc Biol 1995 57 523 528 7722409 10.1002/jlb.57.4.523

[B18] Graesser D Solowiej A Bruckner M Osterweil E Juedes A Davis S Ruddle NH Engelhardt B Madri JA Altered vascular permeability and early onset of experimental autoimmune encephalomyelitis in PECAM-1-deficient mice J Clin Invest 2002 109 383 392 11827998 10.1172/JCI13595 PMC150854

[B19] Duncan GS Andrew DP Takimoto H Kaufman SA Yoshida H Spellberg J de la Pompa JL Elia A Wakeham A Karan-Tamir B Muller WA Senaldi G Zukowski MM Mak TW Genetic evidence for functional redundancy of Platelet/Endothelial cell adhesion molecule-1 (PECAM-1): CD31-deficient mice reveal PECAM-1-dependent and PECAM-1-independent functions J Immunol 1999 162 3022 3030 10072554

[B20] Suzuki K Sugihara G Ouchi Y Nakamura K Futatsubashi M Takebayashi K Yoshihara Y Omata K Matsumoto K Tsuchiya KJ Iwata Y Tsujii M Sugiyama T Mori N Microglial activation in young adults with autism spectrum disorder JAMA Psychiatry 2013 70 49 58 23404112 10.1001/jamapsychiatry.2013.272

[B21] Ashwood P Krakowiak P Hertz-Picciotto I Hansen R Pessah I Van de Water J Elevated plasma cytokines in autism spectrum disorders provide evidence of immune dysfunction and are associated with impaired behavioral outcome Brain Behav Immun 2011 25 40 45 10.1016/j.bbi.2010.08.003 20705131 PMC2991432

[B22] Nelson PG Kuddo T Song EY Dambrosia JM Kohler S Satyanarayana G Vandunk C Grether JK Nelson KB Selected neurotrophins, neuropeptides, and cytokines: developmental trajectory and concentrations in neonatal blood of children with autism or Down syndrome Int J Dev Neurosci 2006 24 73 80 10.1016/j.ijdevneu.2005.10.003 16289943

